# Approaches to assessment in time-limited Mentalization-Based Therapy for Children (MBT-C)

**DOI:** 10.3389/fpsyg.2015.01063

**Published:** 2015-07-27

**Authors:** Nicole Muller, Nick Midgley

**Affiliations:** ^1^Department for Trauma, Attachment Problems and Emerging Personality Disorder, Child and Adolescent Mental Health Service De Jutters, The HagueHolland; ^2^Anna Freud Centre, LondonUK; ^3^Research Department of Clinical, Educational and Health Psychology, University College LondonLondon, UK

**Keywords:** Mentalization, MBT, time-limited therapy, middle childhood, assessment, case formulation, therapeutic focus

## Abstract

In this article we describe our clinical approach to assessment, formulation and the identification of a therapeutic focus in the context of time-limited Mentalization-Based Treatment for Children (MBT-C) aged between 6 and 12. Rather than seeing the capacity to mentalize as a global construct, we set out an approach to assessing the developmental ‘building blocks’ of the capacity to mentalize the self and others, including the capacity for attention regulation, emotion regulation, and explicit mentalization. Assessing the child’s strengths and vulnerabilities in each of these domains provides a more nuanced picture of the child’s mentalizing capacities and difficulties, and can provide a useful approach to case formulation. The article sets out an approach to assessment that includes a consideration of mentalizing strengths and difficulties in both the child and the parents, and shows how this can be used to help develop a mutually agreed treatment focus. A clinical vignette illustrates the approach taken to assessment and connects it to routine clinical practice.

## Introduction

John, 8 years old, has lived with his foster family for 1 year. His carers have contacted our Service because he is aggressive at home and at school. John is socially isolated because he has great difficulty in connecting with other children. The social worker explains that he was placed in foster care at the age of two, and has lived with three different foster families since then. His mother was a teenager when he was born, without a husband and with severe psychiatric problems at that time, which made it difficult for her to be emotionally available for John. During the first individual assessment session, the therapist and John were sitting together, choosing from a table full of various different shells, to explore how he felt within the foster family and also how he felt toward his biological mother, whom he visited regularly. John was strikingly able to place the seashells according to how he saw the relationships in his family. He put the two families, which are both important to him, on their own chairs. He put a shell representing himself balanced between the arm-rests of the two chairs, and told the therapist that he sometimes didn’t know what family he really belonged to. The therapist commented that she could imagine it would be difficult to know where you belong if you have lived in four different families, when you are only 8 years old. John chose the largest shell for his foster father Carey, “because Carey is a very big man.” The therapist linked this to John’s earlier statement that Carey seemed to be very important to John. John agreed enthusiastically.

In this interaction, John is ‘mentalizing’ about himself: He is able to explain how he feels attached to both families but is often unsure where he belongs, using shells to help find a way to express how he thinks about himself in relation to important others. The therapist is being curious about what John is communicating in the way he positions the shells. She really wants to get to know John and is trying to be open-minded about what is inside him and what is happening between them.

John was one of the first children in the time-limited Mentalization-Based Treatment for Children (MBT-C) program, which was set up at the De Jutters Child and Adolescent Mental Health Service (CAMHS), in the Netherlands, from the beginning of 2012. Developing a time limited program started under the pressure of insurance companies no longer wanting to pay for long-lasting therapies. But it was also a response to a growing awareness that, with high levels of mental health needs among children and limited resources available worldwide, there is a pressing need for relatively short-term interventions that are outcome-oriented, and based on a sound understanding of child development and a plausible theory of therapeutic change. However, there are real challenges to developing time-limited ways of working with extremely vulnerable children, such as John. In order to be effective, time-limited work requires a clear approach to assessment, not only to identify those children who are most likely to benefit from this approach, but also as a way of developing a clear formulation that can lead to an agreed focus for the intervention.

In this article we want to describe our clinical approach to assessment, formulation and the identification of a therapeutic focus in the context of time-limited MBT-C. We start by giving a brief introduction to MBT-C, before going on to describe the approach to assessment that has been developed at the De Jutters CAMHS, with a specific focus on the way in which the assessment of the capacity to mentalize, and the way this is linked to the child’s presenting difficulties, can help to create an agreed focus for a piece of time-limited therapy. A number of clinical vignettes will be used to illustrate the approach that has been developed.

## Background

### What is Mentalization-Based Treatment for Children (MBT-C)?

Mentalization-Based Treatment for Children is an adaptation of a therapeutic approach that was developed in the context of adult psychotherapy, in particular for the treatment of adults with Borderline Personality Disorder (BPD, Bateman and Fonagy, 2009). MBT emerged out of a recognition that the key elements of BPD – such as emotional lability, and the instability in personal interaction – could be understood as a consequence of deficits in the capacity to mentalize, i.e., the capacity to be able to understand the behavior of others and one’s self in terms of intentional mental states. Where this capacity is limited or inhibited, interactions with others – as well as one’s own behavior – can often be experienced as confusing and overwhelming, leading to breakdowns in affect regulation and the sense of a coherent self (Fonagy et al., 2002).

Empirical research in the field of developmental psychology and neuroscience has established that the capacity to mentalize is innate to human beings, with its own ‘developmental line’ across the lifespan, linked to specific elements of brain maturation; but that the full development of this capacity is associated with the quality of early attachments and parenting, and that the capacity for mentalizing can be significantly impaired by early maltreatment, abuse and relational trauma (Gergely and Unoka, 2008; Allen et al., 2014; Ensink et al., 2014). It followed from this that a therapeutic approach which focuses on enhancing the capacity to mentalize – at least for adults with BPD – could have significant therapeutic benefits. This hypothesis has now been supported by evidence from clinical trials, and MBT is increasingly recognized as an important new development within the field of adult psychotherapy. In recent years, MBT has been used as an approach to a broader range of psychopathologies, including depression, eating disorders and psychosis (Brent et al., 2014; Luyten et al., 2012; Skarderud and Fonagy, 2012). In recent work, Fonagy and Allison (2014) have suggested that MBT not only addresses the deficits in mentalization that may underlie (or maintain) a range of psychopathologies, but that it also helps to build ‘epistemic trust,’ i.e., trust in the authenticity and personal relevance of interpersonally transmitted knowledge – a crucial capacity which they suggest may be at the heart of all successful forms of psychotherapy.

Although MBT was first developed in the context of adult therapy, from the beginning it was also inspired by the work of child psychotherapists, in particular the tradition of ‘developmental therapy’ that had been established by Anna Freud and her colleagues at the Hampstead Clinic (now the Anna Freud Centre) in London (see Fonagy and Target, 1996; Hurry, 1998). Verheugt-Pleiter et al. (2008) were the first to articulate a model of mentalizing in child therapy, identifying ways in which MBT could be integrated into traditional models of long-term psychoanalytic therapy with children. In recent years there has been a steady growth in the development of mentalization-based interventions for children (see Midgley and Vrouva, 2012), including MBT for families (Keaveny et al., 2012), MBT for Adolescents (Rossouw and Fonagy, 2012), as well as a range of interventions focused on the early parent–infant relationship (e.g., Minding the Baby, Slade, 2006. Although not developed as a distinct model, a number of clinicians have described their own approaches to using aspects of MBT with children (e.g., Ramires et al., 2012; Zevalkink et al., 2012; Lindqvist, 2013; Perepletchikova and Goodman, 2014). The development of a time-limited model of MBT-C at De Jutters in the Netherlands should be understood in the context of this broader set of developments, and emerged out of discussions with a wide range of colleagues (see Lindqvist, 2013), including several from the Anna Freud Centre in London, interested in developments in this field.

The aim of long-term mentalization-informed child psychoanalytic psychotherapy, as described by Verheugt-Pleiter et al. (2008) and Zevalkink et al., (2012), is to enhance mentalization and to strengthen the sense of being able to self-regulate. Separate goals are to enhance a coherent sense of self, to enlarge the possibilities to regulate emotions and to strengthen the sense of self agency. The question is if these goals are also applicable to time-limited work with children. Our initial hypothesis is that working in a limited time-frame means you can help to develop the process in which these goals are set in motion; but for children with more severe levels of disturbance, we would not expect to reach all of these goals by the end of a time-limited intervention. We do not deal with all the problems but we try to promote mentalizing and coping in such a way that a developmental process is back on track, and the family and child feel in a position where they are better equipped to tackle the problems that first brought them to therapy. As Winnicott (1962) put it, the question that one is asking may not be ‘how much can one do?’ but rather ‘what is the least that needs to be done?’ In the case of MBT-C, the aim is to foster sufficient enhanced capacity to be thoughtful about the intentions of the other, and the impact of others on our own mental states, even in the face of stress.

For children seen in the service, the child comes to his MBT-C therapy once a week for 12 weeks, and at the same time the parents are offered MBT-parents therapy. In some cases we work with family therapists as well, who can also visit the family once a week at home as part of our outreach service. After eight sessions we review with the child and his or her parents and decide if we will offer another 12 treatment sessions or if we will stop after 12 sessions. We can prolong up to three series of 12 sessions. The decision to stop or go on with the therapy depends on how quickly the problems diminish and goals are met, and whether the child and family feel that continuing with therapy would help to achieve this. Negotiating this decision is itself part of the work to promote mentalizing, as we aim to explore the situation from multiple perspectives, before making a final decision.

### Which Children might Benefit from Time Limited MBT-C?

The service at De Jutters is for children, age 6–12 years old. Children are usually referred by family doctors, special services for foster and adoption children and therapist colleagues working with adults in psychiatric services, and present with a wide range of difficulties, including internalizing disorders like anxiety problems, post-traumatic stress, and mood disorders but also externalizing difficulties like ADHD, or a combination of both which you often see with reactive attachment disorder. At this stage we are still establishing which children can benefit most from a time-limited MBT intervention. Based on our clinical experience, a time-limited MBT-C approach can be used very effectively with children with mild anxiety problems or mood disorders; however, there are a number of relatively short-term, evidence-based interventions, based on Cognitive Behavioural Therapy (CBT), which have already demonstrated their capacity to support these children (see Fonagy et al., 2014, for a full review), so our focus in developing the MBT-C approach has been elsewhere. Because MBT has a relational focus and is rooted in attachment theory, we think MBT-C is likely to be especially appropriate when attachment relations are at risk; when the duration of the problems is longer and the problems are more complex because of trauma or severe family pathology; or when there is a mix of internalizing and externalizing problems (which may be an indication of emerging personality disorder in adolescence).

It can be challenging to relate these criteria to DSM-5 (American Psychiatric Association [APA], 2013) classifications only. The children who are offered MBT-C in our service are often adopted or in foster care, with histories of chronic trauma, and may have a diagnosis of reactive attachment disorder. Another group of children is from multi-problem families with a parent with a psychiatric disorder. For some children there is a combination of internalizing problems (like anxiety and/or depressive disorder) and externalizing problems, like ADHD and/or behavioral problems, complicated by grief or loss of an attachment figure.

In thinking about which children can benefit most from time-limited MBT-C, we have found it helpful to draw on the distinction made by Fonagy et al. (1993), between children with ‘mental process’ and ‘mental representation’ disorders. In the latter, historically thought of as ‘neurotic’ disorders, the child’s difficulties may be the result of conflicts between different sets of mental representations (e.g., a wish to damage and a fear that to do so would risk the child being rejected by the care-giver). In the former, there may be a more significant deficit in the development of mental functioning itself – possibly for genetic reasons, but in many cases as a result of early trauma and/or abuse. Fonagy (2002) described his way of thinking about children with a severe mental process disorder, who often are severely traumatized, adopted, or foster care children and/or children of parents with psychiatric histories. They seem to have problems such as: an imperfect mental representation of self and others, low frustration tolerance, low self-esteem, no coherent inner world, impaired self-object representation, impaired affect regulation, inflexible defense systems, problems with social capacities, difficulty in noticing the intentions of others, impaired sense of reality, weak attention regulation, or memory function, limited language understanding, especially when this is linked to an emotional context. As empirical studies are beginning to demonstrate (e.g., Schimmenti et al., 2014; Schimmenti and Bifulco, 2015), these children are far more likely to have an insecure or disorganized attachment style. They are the children who are usually referred to our service, and the challenge we set ourselves was to see whether it was possible to develop effective time-limited interventions for this group of children, to whom we might have traditionally offered more open-ended or long-term therapy. In changing our usual practice, we quickly came to appreciate that a careful process of assessment, and a clear development of a therapeutic focus, would be essential if this work was to have any chance of success.

## Discussion

### The Process of Assessment for Time-Limited MBT-C

At De Jutters service, we generally start the assessment phase with a family session, followed by three individual sessions with the child, whilst a separate therapist meets with the parents or carers. The assessment ends with a joint family session, in which the formulation is shared with the child and family, and recommendations are made regarding treatment. The overall aim of the assessment is to develop some kind of ‘mentalizing profile’ of the child, parents, and family, and to explore what links this might have with the difficulties that brought the child to treatment. If the child is offered time-limited MBT-C, we also use the assessment process to help reach an agreed focus for the work. At the same time, we hope that the assessment process is therapeutic in itself, and also allows us to assess the child and family’s capacity to make use of this particular way of working.

#### Meeting the Child and Family for the First Time

The first family meeting – which both therapists usually attend – is somewhat structured, and draws on ideas developed in the context of family-based MBT (Keaveny et al., 2012). The aim of the initial family meeting is to try and build an initial alliance with the family, and introduce them to some of the key components of a mentalizing approach – but also to help us understand something about the quality of attachment relationships in the family, and to identify strengths and weaknesses in the family’s capacity to mentalize together, including any specific areas (e.g., when issues of aggression or sexuality are raised) where the family’s capacity to mentalize appears to be vulnerable.

There are three elements to this initial family meeting: first, each member of the family is invited to introduce one of the others, and is asked to say a little bit about them as a person (e.g., what they like, or the kind of person they are). After this verbal introduction of each other we ask the family members to select an animal for each other, and invite some discussion about these choices, looking for more implicit information about how different members of the family see each other or see themselves. There can often be a playful quality to these introductions, as family members surprise themselves (and each other) with the way they are introduced, or the images that others have of them. We then ask all the members why they think they are here, and listen to their own problem formulation, which is important information to keep in mind when looking for a focus of the therapy. After this we finish the session by doing a family game, which gives the therapist the opportunity to see how the family members interact with each other. Often we choose a structured task, when some structure is needed; because we want it to be a good experience for everyone and not to let things get out of hand. However, when the family has already shown some capacity to take turns, listen and work together collaboratively, we like to choose a freer task like drawing or creating something out of clay together. This might involve us inviting them to make their dream family house, or a family zoo. One reason for doing some type of family activity is to see how the family relate to each other in a play-based situation – the process of doing this is as important (if not more so), than what is actually created.

During our first family session John was there with both his foster parents. When he had to choose an animal for each person, John chose a chimpanzee for himself. John’s foster mother guessed this was because he could sometimes be quite cheeky. When the therapist checked this with John, he nodded, but added that chimpanzees also have sharp teeth. The therapist wondered aloud why that mattered to John, and John’s foster father said that chimpanzees sometimes had to protect themselves when things didn’t feel safe. John smiled, and then chose a gorilla for his foster father and he pointed out that they are both monkeys, but from different families. “They belong to the family of the apes, they belong to each other but they also have their own family,” John said with a lot of feeling. Foster father responded to him in a warm and genuine way, putting his arm on his shoulder in a gentle way, and adding, “Just like us.”

#### Assessment Meetings with the Parents

The three sessions with the parents usually take place while the child is being seen by a separate therapist, and in these three sessions the therapist tries to use the core features of the ‘mentalizing stance,’ including empathy, curiosity, and an interest in different perspectives, to try to get an appraisal about the problems and also about the mentalizing capacities and difficulties of the parents themselves (see also Muller and ten Kate, 2008; Muller and Bakker, 2009). By that, we mean the parent’s capacity to think of the child as a separate person, with a mind of his or her own, and to see the child’s behavior (and their own, as parents) in terms of intentional mental states (Slade, 2006). Although we do not explicitly make use of Slade’s Parent Development Interview (PDI, Slade et al., 2004), we find some of the questions that are used in this attachment-focused interview helpful clinically, such as ‘I’d like you to choose three adjectives that you feel reflect the relationship between you and (your child). Does an incident or memory come to mind with respect to each of these adjectives?,’ or 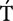
ell me about a time in the last week or two when you felt really angry as a parent. What kinds of situations make you feel this way? How do you handle your angry feelings? (see also Muller et al., 2012). This helps to open the conversation to feelings parents might feel ashamed of. Where appropriate, we might also ask a question that is part of the Adult Attachment Interview (George et al., 1996): ‘How do you think your own experiences of being parented affect your experience of being a parent now?’ This question helps us to identify possible ‘ghosts in the nursery’ (Fraiberg et al., 1975), i.e., issues or experiences from the parents’ own histories which may be influencing the way they relate to their child.

Slade et al. (2004) have also developed a coding of the PDI interview for ‘Parental Reflective Functioning’ (i.e., the specific capacity to mentalize in relation to one’s child), and although we do not use this coding system in any formal way, we have found this helpful to alert us to key features of parental mentalizing (or failures of mentalizing). For example, we look to see whether the parents show a curiosity about exploring the meaning of their child’s behaviors in terms of intentional mental states (e.g., whether his difficulty with separation might be related to his worries about his mother’s health difficulties); and whether they are able to acknowledge the ‘opacity’ of their child’s mind, i.e., that they can guess what their child may be thinking or feeling, but that none of us can ever be absolutely certain what is happening in the mind of the other. Features like this are helpful indicators of the parent’s capacity to sustain a stance of ‘mind-mindedness’ (Meins et al., 2002), even in the face of their child’s difficult behavior.

In the assessment of the foster parents they underlined the importance of healthy food. After John had visited his mother he told stories about all the candy and the snacks he had eaten. Back in the foster family he had trouble eating the normal food, which irritated the foster parents a lot because he spoke extensively about everything he ate when he was with his mother. They explained to him that the food of his mother was not really healthy and the food in their family was, and they instructed him not to eat too much candy while visiting his mother, because of his weight problems. But after this they explained that John stopped telling them about what he ate when he visited his mum, although they guessed he was probably still eating a lot of candy. John’s foster mother spoke of her frustration about this, and said she couldn’t understand why John didn’t listen to their advice. After further exploration, it became clear that foster mother had problems with being overweight herself when she was a child, and was very worried that John would be bullied in the way she had been. Her voice became softer as she remembered her own experiences, and the therapist then invited them to think whether John’s reasons for eating the junk food might be similar or different to foster mother’s, when she was a child. The foster carers began to think about how, for John, what he ate might feel like a question of which ‘parents’ he was loyal too. Feeling sympathy with his situation, they thought about whether they should stop talking to Jack about the unhealthy food and instead react positively to his enthusiastic way of telling, and to respond to his happiness about the contact with his mother. They decided to try and change their way of speaking to John, wanting to emphasize instead that there are differences in the way things go in the two families, but that it was okay to enjoy these differences. Foster mother in particular recognized that this wouldn’t be easy for her, but that this was more her problem than John’s.

In this assessment meeting with John’s foster carers, it was apparent that they were able to use the space provided by the therapist to explore John’s experiences, and to separate their own needs and wishes from his. In doing so, they were able to think how they could best provide him with a sense of security and safely. This is not always the case with the families who are referred to our service. As part of the assessment meetings with parents, we always try to assess if the parents, or the home-situation is (to use Winnicott’s term) “good enough.” This topic is a source for team discussion, because many of these children come from multi-problem families. But when there is no safe haven an intervention to take the child out of the threatening situation might be necessary before starting therapy.

#### Assessment Meetings with the Child

Alongside our meetings with the parents, we do an assessment of the child, usually in three sessions, and try to make a profile about the global functioning of the child, in which a link is made between the child’s capacity for mentalizing and his or her presenting problems and difficulties. We also try to look for the vital spark in the child (Winnicott, 1971); something that seems a strength or a little burning flame that might help in the therapy. It can be things he likes to do or is good at or what he is curious about. We also contact the school to hear their thoughts, problems and observations about the child. Although we do not routinely use validated, structured measures as part of the assessment, these can be incorporated. For example, some referrals might require a cognitive assessment, such as the Wechsler Intelligence Scale for Children (WISC, Wechsler, 2004), or the use of a screening tool for autistic disorders, such as the Autism Spectrum Screening Questionnaire (ASSQ, Ehlers et al., 1999). These can be incorporated, according to clinical need, as long as they are conducted in a manner consistent with the overall therapeutic stance (see below).

Each assessment session usually lasts for approximately 45 min, and the sessions are relatively unstructured, although the therapist will provide a set of objects that the child can use. In these sessions about 30 min are free play for the child and 15 min are structured by the therapist. We always make time to create a genogram using shells or animals, which sometimes may last a bit longer than 15 min. The child is asked to pick a shell for each family member, and place them to describe the family. The choosing and placing of shells can be very revealing of the child’s view of the family and a good starting point for talking about representations of self and others, now and retrospectively. We always try to do a little bit of projective research in asking the children to tell a story using several pictures, such as those used in the Thematic Apperception Test (TAT; Murray, 1943). And we include some drawings which are to be finished by the child, trying to see what they know about how emotions look. In line with the MBT approach, during these sessions the therapist should feel free to be active and responsive and engaging in play if that is regarded as helpful. Because many children with attachment disorder are chaotic and fragmented we often choose not to work in the playroom, because of the overload of toys and the size of the room, but to work in a more contained little office with limited play materials.

The ‘stance’ taken by the therapist in MBT-C work is absolutely essential, especially when we are working with chronically traumatized children where the theme of limits is all the time an explicit and implicit theme in the therapeutic relationship and the therapy. These children often cannot find words to express their dissociated or denied experiences but often evoke these feelings in others, in enactments. Therefore it is essential for the child psychotherapist, just like the sensitive and supportive parent, to continually pay attention to his or her own subjective experiences, what is felt in the relationship with the child, the non-verbal language of emotion and in the own body (Wallin, 2007). This is what calls affect attunement Stern (1985, 2004, in Wallin, 2007, p. 60), which means being present, participating, moving along, sharing the subjective experience of someone else, without trying to change them. In this way you can give meaning as a child therapist to the expressions of a child, without knowing, without wanting to change, but by absorbing, tolerating, bearing the feelings of the child trying to understand, to attune and to be curious.

By mentalizing about the therapeutic relation and searching for what cannot be articulated, from ‘mismatch and repair’ (instead of a perfect understanding), we search for hypotheses about the inner world of the child. The focus is mainly in the here and now of the therapeutic relationship. The therapist tries to convey to the child that she is someone who wants to help, and that she is interested in the experiences of the child. Confirming the child’s gestures, thoughts, and feelings, as well as exploring the child’s intentions, the therapist aims at strengthening the child’s self-agency. The therapist is following the child in the content of the play but is active in managing and creating the process.

In the assessment phase we try to find out, based on our clinical experience and understanding of child development, if a child is functioning according to his or her developmental age and explore the child’s interests, longings and friendships. But we are also paying particular attention to the child’s capacity to mentalize. In thinking about the assessment of the capacity to mentalize in middle childhood, we do not currently use any formal research assessment, but we have found it helpful to draw upon the work of ?, who have developed a Child Reflective Functioning Scale (CRFS), which has been used to rate children’s responses to the Child Attachment Interview (CAI, Shmueli-Goetz et al., 2008). This is an adaptation of the Adult Attachment Interview (George et al., 1996), the most widely used measure of adult reflective functioning. As with the PDI for parents, a number of questions in the CAI can be helpfully drawn on as part of a clinical assessment (e.g., ‘Can you tell me three words to describe your relationship to your father?,’ ‘Can you tell me about a time with your dad that links to each of those words?’); but we have used the CRFS primarily to help alert us to some of the features of mentalizing in middle childhood that indicate areas of strength and vulnerability in the child. For example, the CRSF highlights certain features of the capacity to mentalize the other in middle childhood, such as the ability to understand that different people may perceive a given behavior differently, based on their knowledge or beliefs; but it also gives helpful indications of what we might look for when this capacity is impaired, such as bizarre responses, or descriptions of behavior without any reference to mental states (‘mum did this and then she did that and then I did this’). Watching out for some of these ‘mentalizing markers’ in the course of the assessment can be very helpful, when trying to make a formulation.

Rather than seeing the capacity to mentalize as a global construct, we have found it very helpful to think about the developmental ‘building blocks’ of explicit mentalizing, as set out by Verheugt-Pleiter et al. (2008). In this work, they distinguish between attention regulation, emotion regulation and mentalization (Verheugt-Pleiter et al., 2008). Assessing where the child is in relation to each of these elements provides a more nuanced picture of the child’s mentalizing capacities and difficulties.

In exploring the attention regulation capacities of a child, we ask ourselves a number of questions as the assessment progresses. Can the child regulate his impulses, focus his attention, listen to others, behave according to his developmental age? Does the child have a sense of a skin in which he lives? When there has been some containment in the early phase of life a child learns he has a skin, which is a natural limit of his body and at the same time is the beginning of a sense of an internal and external world. It is the basis of a normal sensori-motor regulation capacity and the integration of bodily, posture and movement experiences. Therefore we always try to look for striking reactions to sound, light, touch, temperature, movement in space, gross motor skills, and fine motor skills. Is a child open to everything around him, overly aroused, or hypersensitive?

During his assessment meetings, John reacted very sensitively, particularly to sound. He heard others talking in the adjoining room, or the slamming of doors and was immediately distracted. About his fine motor skills the therapist noticed that he used the tweezers grip and was reasonably well able to work in a fine motor way, which he demonstrated in the coloring of some drawings. He was not yet able to write the letter J of John, his own name; he wrote the little hook at the bottom of the letter J in the wrong direction. If he took pleasure in an activity he was well able to keep his attention span. If he found an activity difficult (like trying to compose a small story) he made it very clear that he didn’t like it. He was then much less able to maintain his attention span. John was sufficiently well able to regulate his attention, and he could also enter into moments of joint attention. During emotionally charged moments, like during the making of the genogram with the shells, it was immediately clear how little he had learned about focussing and maintaining attention about what he felt inside. With the help of the therapist he could begin to find some words to express himself a little bit. But he then got very distracted and active and stepped out of the contact with the therapist, doing something on his own.

When a child has a difficult start in life or lives in difficult circumstances he can often only poorly mentalize about painful or vulnerable matters and will often act them out through his body. Such children are often easily aroused and hyper-vigilant, which makes it harder to regulate their attention and emotions. Being able to manage impulses from the inside is an essential requirement that precedes learning to mentalize, because a mental representation has to get more priority than the physical reality (Verheugt-Pleiter et al., 2008). In other words, you have to be able to endure a feeling without immediately action to be able to mentalize.

In order to get some indication about the emotion regulation capacity of the child we want to know which emotions the child knows? Which emotions are a problem? What are the antecedents of a problematic behavior or feeling? We might read a book with the child in which all kinds of feelings are drawn using several fish drawings and ask the child if he can recognize how the fish feels. We then ask the child to draw a fish himself. We also show some pictures and ask the child to make up a little story and try to link this little story to his own life. Is a child able to play or fantasize or not? Does the child accept limits? What enactments does the therapist see or sense in the room? What are the therapist’s own feelings and thoughts about this child?

In the second session John and his therapist were working with clay and his work got stuck to the table. Therefore he had to start again because it was not possible to remove the clay work from the table without damaging it. Despite the frustration, he showed he was very well able to accept limits. Limits in time, limits in material. He indicated that he took pleasure in playing with water during clay sculpting, but he refrained from doing this right away and by seeking eye contact with the therapist he asked for approval first. When he was emotionally touched by a topic, like when placing the shells, he had difficulty saying why it was touching him or what happened inside him. He demonstrated that it emotionally touched him by wanting to do something else, standing up, or starting to talk about something else. When the therapist underlined his behavior by saying it might be painful for him not knowing where he belongs he didn’t show a reaction. When he saw a seashell in a corner of another table he briefly mentioned that this must be a naughty shell that needed to lie in the corner. When the therapist asked him if he himself sometimes had to stand in a corner because he was naughty he nodded sadly.

To monitor the explicit mentalizing capacities we want to know if a child has any representation of him- or herself? Of others? Any attunement toward others? Any curiosity toward others or himself? Any fantasy? Drawing on a question from the CAI, we ask for three words to describe himself, and then we ask for an example of each adjective (e.g., can you tell me about a time when you were ‘angry?’). We try to look at whether the child has a capacity for explicit mentalizing (i.e., to be curious about his own or other’s thoughts and feelings, and how they might relate to the way he or others behave), and if he does, we try to explore in what contexts the child is able to make use of this, and in what contexts such a capacity breaks down. This is important because mentalizing is not a fixed capacity, but comes and goes, according to the context and our levels of emotional regulation.

During the second session John wanted to play with the clay. He started making a bowl for his mother, in the shape of a heart, because he said he loves her very much. He then wanted to make a Donald Duck bowl for his younger brother (who lived with their mother), but while working on this he started thinking that the heart bowl could very well be for everyone. He made a second bowl for his foster father because he says he loves him too very much and that bowl becomes a cat’s bowl. Then there was some clay left, and John spontaneously came up with the idea that he would like to make a bowl for himself. He wanted to make the Donald Duck bowl for himself and not for his brother. The therapist thought to herself that it was difficult for John as his brother lived with their mother and he probably had jealous feelings about it, at least according to his foster mother. John told her that the bowl he was making for himself must be very strong. He reinforced the edges of the tray well. “It must be able to hold water, that is very important,” he told her. The therapist wondered aloud if he wanted to become strong himself, not being angry all the time, being able to keep his feelings inside him. John nodded, and said that he worried his foster parents wouldn’t want to keep him when he got angry all the time.

#### Gathering Our Thoughts into a Formulation – And Creating a Story

The fifth and final session in the assessment phase is again with parents and the child together, where we present our assessment in a way that we hope can be understood by both parents and the child. In this meeting we try to formulate something about how we have mentalized about the child, the family and how these link to the problems that brought them to treatment.

In the case of John, our assessment indicated that John’s foster carers have a good capacity to see John as a boy with thoughts and feelings, which can help them to make sense of his behaviors. Although there were areas where their capacity was limited, possibly due to issues deriving from their own histories (e.g., foster mother’s response to John not being careful about his diet and putting on weight), they were able to make use of the therapeutic space provided in the assessment to reflect on their own mental states, and thereby separate out what belonged to John and what belonged to them. In doing so, they were able to think about John’s loyalty conflict in a different way, and modify how they responded to his behaviors accordingly.

Likewise, John demonstrated a capacity, under the right circumstances, to regulate both his attention and affects, and even to make use of explicit mentalizing to make sense of his own behavior and the reactions of others (i.e., when he spoke about his worry that his foster carers would reject him if he behaved badly). However, it was also apparent that these capacities could easily be lost or compromised when John felt anxious or afraid; and that John’s vulnerability to such disruptions were probably heightened by his early history, during which he may not have received the kind of ‘maternal mind-mindedness’ (Meins et al., 2002) or ‘contingent mirroring’ (Fonagy et al., 1993) that helps to develop the child’s own capacity to mentalize. John’s reported aggression and social isolation could be understood as a consequence of this vulnerability, and it was felt that a short-term MBT-C intervention, with work with his foster-carers alongside it, could help to begin him to strengthen these capacities, in the context of a therapeutic relationship in which he was able to gradually regain a sense of ‘epistemic trust’ (Fonagy and Allison, 2014).

Sharing such a formulation with children and their parents is not always easy, and it is important to avoid using overly technical language. We have recently experimented with offering the family a small story, which offers them some of our own thoughts about what we have learnt from the assessment, using a metaphorical language which invites the family to engage with the work at a symbolic level.

In their final session, the therapist thanked John and his foster carers for coming, and explored how they had found the process so far. She then told them that she’d been thinking a lot about all they had shown and told her, and that she had made a small story, which she would like to share with them. John looked very pleased about this, and he leant in against his foster mother’s body, as if waiting to hear a story at bed time. The therapist then began: There was once a little chimpanzee. He lived for a while with his mother in a group but had to leave her when he was really little, because she couldn’t take care of him anymore. That was sad, because the chimpanzee hadn’t learned all the words and rituals that are used in a chimpanzee family. After traveling around, and staying in different places, the little chimpanzee came to a family of gorillas. This looked a bit like home, but sometimes he felt out of place and worried if the gorilla family would let him stay. He often lacked the words to describe what he thought or felt. He sometimes felt very alone because he missed his mother and because he had lived so long with others where he had felt an outsider. When he felt sad he sometimes became angry, because that helped him feel a bit bigger. At the gorilla’s it did feel like home often, but sometimes it didn’t. He was a chimpanzee after all. So he decided he wanted to find his own words and rituals to become stronger and not so angry anymore and he decided he wanted to live with the gorillas and visit the chimpanzee family once in a while. The gorillas loved the little chimpanzee and they were willing to learn more about how a chimpanzee is.

### On ‘Focus’

Inspired by Developmentally Directed Time Limited Psychotherapy for Children (Haugvik and Johns, 2008) core features of arranging the therapy constellation of a time limited MBT-C program are choosing a focus or metaphor for the therapy, which should emerge out of carefully observing and listening to everything the child conveys during the assessment sessions, both verbally and non-verbally. This focus can be helpfully summed up with a motto, or short phrase, which can be shared with the child and parents at the start of treatment. Together we try to look for a motto which gives meaning for the therapy and in which the child feels confirmed and recognized. Often the motto will draw upon something the child has said or drawn, so that it is a joint creation, between the therapist and the family. Sometimes the child doesn’t come up with anything to contribute to the motto. The metaphor can still be formulated because it helps the therapist to focus and mark the playing field for the therapy and it usually helps the parents. Sometimes the therapist spontaneously looks for a motto together with the child in the last assessment session. But more often the therapists think of a motto or little story and share this with the child and parents in the final assessment session.

The mutual treatment focus or metaphor represents a time perspective and can be related to Stern’s concept of “key metaphor,” representing relational and emotional central themes (Haugvik and Johns, 2008). The focus becomes a joint point of departure as well as a direction for the therapy, and is an important part in forming the therapeutic alliance. In the focus, the therapist helps the child to know what is going to happen in the therapy. Many children are sent to therapy by parents or other adults, and do not know why they have to come. When time is limited the task of stimulating agency and participation in the child is extra important. The focus functions as an invitation to the child to engage in the therapy process.

It is important that the focus bears meaning to the child, creating an experience that “this is about me.” The therapist conveys by the focus that he or she is someone who wants to help the child and who knows about the child’s difficulties. The starting point in focus-formulation is the reason for the family seeking help. However, the focus must not be a problem description. Rather it is supposed to be a support to the therapeutic direction by pointing to the child’s resources and skills, and what can be developed in therapy. It also stimulates parental reflective functioning by directing the parent’s attention to the child’s inner world. Furthermore it helps the child’s mentalization by conveying that he or she is held in mind, and stimulates the child to be interested in their own feelings and thoughts. This way the focus becomes a model for both parents and child for how one can “hold someone’s mind in mind.” The focus also helps the therapist, directing him to a mentalizing approach toward both parents and child.

John was touched by the story about the chimpanzee and the gorilla family; he became quiet and looked seriously at the therapist. He then spontaneously said ‘I want to become a proud and happy chimpanzee living in peace with other apes.’ The therapist asked John if this might be a focus for the therapy? John thought that would be a good idea. ‘We have work to do,’ the therapist said to John, with a smile. She then checked with the foster parents, who agreed on the focus, pointing out they had work to do as well, because they wanted to find ways to help the chimpanzee to feel more in control of his emotions and more connected to them and the other family members. When everyone agreed on the focus the child therapist and John went to their own play therapy room and John made a little monkey out of clay with a big smile on his face. The therapist made such this was put in the room on the same spot, every time John came in for his therapy, to help them keep the therapy focus in mind.

By using material from the initial sessions the therapist can formulate a focus that resonates for both child and parents. Counter-transference feelings can capture relevant themes as well as feelings that the parents might have toward the child. Using these feelings in the focus can be helpful in bringing curiosity of both parents and child, but can also alert the therapist to places where their own mentalizing capacity may have become temporarily inhibited. For example, when reviewing her feedback session in supervision, John’s therapist noticed that the focus that she had proposed spontaneously during the meeting (‘I want to become a proud and happy chimpanzee living in peace with other apes’) focused more on desired changes in his behavior (and feelings), which perhaps reflected a pressure to try and sort out John’s difficult behavior. Perhaps something more exploratory, such as ‘Getting to know the chimpanzees and the gorillas better,’ or ‘Finding out what chimpanzees need to be proud and happy,’ would have encouraged more of a sense of curiosity and interest in mental states and their link to how we feel and behave?

The child’s own use of metaphors related to its experiences in play can be helpful in formulating the focus in a way that the child can relate to. The focus should be short, easy to understand for both child and parents, and demonstrate recognition as well as curiosity and hope. Themes of the focus are often related to control, autonomy, dependency or self-esteem. The focus formulation is exploratory and/or affect regulatory. Furthermore it needs to raise recognition and give meaning to the parents (Lindqvist, 2013).

## Concluding Remarks

The capacity to mentalize is a spontaneous, intuitive and unconscious process, which most of us use all the time without noticing, in order to help us make sense of the way others behave, and the way we respond to the people in our lives. But just as all of us are prone to context-specific losses of this capacity, especially in stressful circumstances, so too more entrenched mentalizing difficulties can often underlie – or at least help to maintain – a range of mental health difficulties that may bring a child to the attention of services. A time-limited MBT-C intervention could be one approach to helping these children and their families. Although the approach still requires systematic empirical validation, the model is consistent with empirical research on the role of mentalizing in psychopathology, and addresses problems that are well recognized among clinicians who deal with traumatized children. Based on our clinical experience so far, we are very optimistic about how well this time-limited MBT-C protocol can work for a range of children – although we don’t yet know who actually benefits most from this approach, and research systematically evaluating the effectiveness of this approach, as well as identifying ’what works for whom’ – will be essential.

In this article we have described the assessment process that we have developed in a service offering time-limited MBT-C. It is always important to do a sound assessment of the child and the parents to be able to choose a focus within a time limited frame. An assessment that focuses specifically on both evaluating and promoting the capacity to mentalize has a number of advantages. When the parents’ capacity to mentalize increases it helps enormously to stimulate the mentalizing capacity of the child. Working at the same time with the parents and child enhances the therapeutic process. Supervision meetings with other child therapists and the family therapist together can help to unravel enactments and help ensure that the therapist notices her own breaks in mentalizing. An open curiosity about such breaks in mentalizing – whether in oneself or others – is at the heart of a mentalizing approach to therapy.

At the end of two phases of MBT-C (i.e., 24 sessions in total), John learned to be able to recognize, name and express more about all he felt about his life in the here-and-now: his loyalty toward his mother, his jealousy toward his biological brother and his two foster sisters. He learned to express his anxiety about having to leave his foster home again, his difficulty of really trusting and believing he could stay with his foster parents and was loved for who he was. He also put into words his anger about having to leave his biological family and two other foster families. Most of the time he could tolerate difficult feelings inside him instead of acting them out. He felt he was stronger and had more self-agency. This led to changes in the way he interacted with others in the foster family, with his biological mother and at school with peers. For the first time he was invited to a birthday party of a classmate at school. He had the feeling he was becoming who he really was, without the persistent aggressive outbursts and mis-trust toward others. Although his interactions with his foster carers was not always easy, by the end of the time-limited MBT-C John and his foster carers knew much more about the chimpanzees and gorillas – what made them frightened or happy, in what ways they were similar, and in what ways they were different.

## Conflict of Interest Statement

The authors declare that the research was conducted in the absence of any commercial or financial relationships that could be construed as a potential conflict of interest.
